# Survival after cancer in children, adolescents and young adults in the Nordic countries from 1980 to 2013

**DOI:** 10.1038/s41416-019-0632-1

**Published:** 2019-11-13

**Authors:** Klaus Rostgaard, Henrik Hjalgrim, Laura Madanat-Harjuoja, Tom B. Johannesen, Sofia Collin, Lisa L. Hjalgrim

**Affiliations:** 1 Department of Epidemiology Research, Statens Serum Institut, Copenhagen, Denmark; 2 Department of Haematology, University Hospital of Copenhagen Rigshospitalet, Copenhagen, Denmark; 3 Dana-Farber Cancer Institute/Boston Children’s Cancer and Blood Disorders Center, Boston, MA USA; 4 Finnish Cancer registry, Helsinki, Finland; 5 Registry Department, Cancer Registry of Norway, Oslo, Norway; 6 Department of Evaluation and Analyses, Epidemiology and Methodological Support Unit, National Board of Health and Welfare, Stockholm, Sweden; 7 Department of Paediatric Haematology/Oncology, The Child and Youth Clinic, University Hospital of Copenhagen Rigshospitalet, Copenhagen, Denmark

**Keywords:** Cancer epidemiology, Prognosis, Paediatric research

## Abstract

**Background:**

The present study aimed to assess whether the widespread concern of inferior cancer survival in adolescents and young adults (AYAs) compared with children and adults holds true in a Nordic setting with important differences in healthcare organisation compared with the United States (e.g. free access to healthcare) and the United Kingdom (e.g. young teenagers are treated in paediatric departments).

**Methods:**

Five-year relative survival was calculated for 17 diagnostic groups in patients diagnosed in 2000–2013 in three diagnostic age categories: children (0–14 years), AYAs (15–24 years) and adults (25–34 years).

**Results:**

For 13 out of 17 diagnostic groups examined, there was no difference in survival between AYAs and neighbouring age categories. For acute lymphoblastic leukaemias, astrocytomas, rhabdomyosarcomas and non-rhabdomyosarcoma soft tissue sarcomas we found survival in children to be superior to that in AYAs. For these four diagnostic groups, the rate of survival improvement over three calendar periods (1980–1989, 1990–1999 and 2000–2013) was not particularly low in AYAs compared with neighbouring age categories.

**Conclusions:**

The present study suggests that in an affluent setting with free access to healthcare, meaningful differences in survival between AYA patients and either childhood or adult patients are a phenomenon of the past for most AYA cancer diagnostic groups.

## Background

Each year, ~1000 adolescents and young adults (AYAs), age 15–24 years, are diagnosed with cancer in the Nordic countries.^1^ In addition to the related morbidity, these cancers also carry a considerable mortality (in the order of 120 deaths annually), corresponding to a substantial number of life years lost due to the patients’ long life expectancy.^1^

Cancers in AYA are mostly of the same types as those occurring in younger age groups, although early-onset adult cancers are also seen, e.g. some carcinomas.^2^ Nevertheless, recent studies from the United States and United Kingdom have indicated that survival of AYA cancer patients may be inferior to that of children with the same disease,^2–4^ and that the improvement in treatment outcome achieved for children with cancer in recent decades is not visible in AYA patients.^2^

The prospect of AYA patients being disadvantaged with respect to survival and other quality-of-life indicators has spurred a search for both explanations and solutions on top of statistics characterising the problem.^3,5^ Explanations include (1) that the age group lies in the traditional organisational divide between paediatric and adult oncology departments and that their treatment therefore is not standardised,^5,6^ (2) that the needs of AYA cancer patients differ physiologically (puberty) and psychologically from both children and older patients,^7^ (3) that AYA cancer patients may also experience longer diagnostic delays due to misinterpretation of symptoms by both the patients themselves and by healthcare professionals^7^ and (4) that the AYA group as such is less inclined to participate in and comply with clinical trials than other age groups, leading to slower and less successful development of new treatments.^3,4,8–12^ Attempted solutions include (1) specialised care for the AYA patient group,^7^ (2) more collaboration among paediatric haematologists/oncologists and adult haematologists/oncologists to make common treatment guidelines and protocols,^3^ (3) expanding the age range of paediatric treatment protocols, as it seems to increase survival for the AYA group to be treated according to these^13,14^ and (4) new work packages aimed at AYAs.^9^

To further gauge the need for such efforts, the present study aimed to assess whether the observed patterns of inferior AYA survival compared with children and adults also apply to the Nordic setting with important differences in healthcare organisation compared with the United States (e.g. free access to healthcare) and the United Kingdom (e.g. young teenagers are treated in paediatric departments).^2,15^ We therefore hypothesised inferior survival for AYAs with cancer compared with children (0–14 years at diagnosis) and adults (25–34 years at diagnosis) in the calendar period 2000–2014 in the Nordic countries within groups of common AYA cancer. As an explanation for such current inferior survival, we further hypothesised inferior improvement in survival for AYAs with cancer compared with children and adults in the calendar period 1980–2014 within the main groups of AYA cancer. We chose age categories optimised to examine cancer survival in age categories that could probably be subjected to childhood cancer treatment regimes, rather than the very broad AYA age range adopted by the most recent large study in accordance with recommendations from the US National Cancer Institute and the European Network for Cancer in Children and Adolescents.^5^

## Methods

Information on all incident cancer cases diagnosed at the age of 0–34 years was retrieved from Sundhedsdatastyrelsen, Denmark (1980–2013); Socialstyrelsen, Sweden (2000–2013); Cancer Registry of Norway (1980–2013) and Finnish Cancer Registry (1980–2013). Cancer registration is nation wide with high completeness in all four countries.^16^ Cases were classified into diagnostic groups according to the international Classification of Childhood Cancer, Third Edition (ICCC-3) by using ICD-10 and ICD-O-3 codes.^17^ All cases in 6 main groups and 29 subgroups of the ICCC-3 were extracted. All cases within each of these 35 groups were further grouped according to country, sex, age (0–14, 15–19, 20–24, 25–29 and 30–34) and calendar period (1980–1989, 1990–1999 and 2000+) at diagnosis. Each case was followed up for death from any cause from time of diagnosis to death, 1 January 2014 (Denmark, Finland and Sweden), 1 January 2015 (Norway) or 10 years past diagnosis, whichever occurred first. There was no loss to follow-up and cases diagnosed at autopsy were excluded. The follow-up of each case was divided into intervals of years since diagnosis with lower limits (0, 0.5, 1, 1.5, 2, 2.5, 3, 4, 5, 6 and 8). For each such cell, we counted years of follow-up, number of deaths and expected number of deaths as well as the number of persons contributing follow-up time. The expected number of deaths was calculated as the sum of products of follow-up time and the matching background mortality rate according to country, sex, calendar year and 1-year age group. The background mortality rates were obtained from the Human Mortality Database.^18^

The aggregated data for analysis specified above were extracted by us from individual deidentified cancer registry records from Norway and Finland, generated from Danish individual deidentified person records at our Research Server at Forskerservice, Sundhedsdatastyrelsen in Copenhagen and generated from Swedish individual person records by National Board of Health and Welfare in Stockholm by a simple adaptation of two SAS programmes used to extract the other data. Cells in the aggregated data with contributions from less than five persons (Denmark) or three persons (Sweden) were excluded.

We used Poisson regression to model piecewise constant hazard functions^19^ comprising fixed background mortality rates and modelled excess hazard rates of interest.^20^ These modelled excess hazard rates were used to construct cumulative hazard functions yielding 5-year relative survival as our effect measure.^20,21^ Throughout we represent relative survival in %. The models were fitted by using the HPNLMOD procedure in SAS, providing easy access to estimators and their standard errors through its predict logic. Confidence intervals and tests were based on Wald statistics. All data processing was performed with SAS (SAS Institute, Cary, North Carolina) version 9.4.

In all analyses, patients were stratified according to age at diagnosis as children (age 0–14 years), AYAs (age 15–24 years) and adults (age 25–34 years) with AYA as the reference category. The main analysis was based on patients diagnosed in calendar year 2000+. For a subset of diagnoses, we analysed the temporal development over three calendar periods (1980–1989, 1990–1999 and 2000+) at diagnosis. The latter analyses were based exclusively on data from Denmark, Finland and Norway. A diagnostic group was only analysed subject to there being ten or more deaths observed within 5 years of diagnosis in the relevant age and period stratum. The working hypothesis was that of general inferior survival for AYA cancer patients compared with neighbouring age categories. In order to assess such a general trend that may be overlooked due to limited statistical power when assessing one diagnostic group at a time, we also took a direct standardisation approach and calculated the overall 5-year relative survival in a hypothetical standard population with number and type of cancer cases as in the combined AYA and childhood population of analysed diagnostic groups, when subjected to AYA survival patterns and childhood survival patterns, respectively. That is, we calculated RS5_AYA_ = Σ_i_ (RS_AYA,i_ × n_i_)/(Σ_i_ n_i_) and likewise with the same weights n_i_ for the ith diagnostic group for children, to have an overall comparison undisturbed by case mix. Likewise, we compared overall 5-year relative survival between AYA and adult cancer patients.

## Results

Comparisons of relative 5-year survival after cancer between persons diagnosed as children (age 0–14 years), AYAs (age 15–24 years) and adults (age 25–34 years) are presented for 17 non-overlapping diagnostic groups commonly encountered in AYA oncology (Table 1). For four diagnostic groups (acute lymphoblastic leukaemia (ALL), astrocytoma, rhabdomyosarcoma and non-rhabdomyosarcoma soft tissue sarcoma (NRSTS)), we found a statistically significant difference (*p* < 0.05) in relative 5-year survival between AYA and at least one neighbouring age category, and applying a stricter Bonferroni corrected criterion (*p* < 0.05/27) only removed rhabdomyosarcoma from this list of potentially interesting diagnostic groups. In all four diagnostic groups, children experienced a better 5-year survival than AYAs, and among astrocytoma patients, AYAs had superior 5-year survival compared with adults (Table 1). Five-year relative survival with 95% confidence interval in a standard population comprising the analysed diagnostic groups common to children and AYAs were 83.4 (82.3–84.5) for children and 74.4 (72.4–76.4) for AYAs, the difference being statistically significant (*p* < 5 × 10^−15^). However, restricting this standard population by excluding the four atypical diagnostic groups yielded a 5-year relative survival of 79.2 (77.4–81.1) for children and 76.8 (74.4–79.2) for AYAs, the difference no longer reaching statistical significance (*p* = 0.12). A similar comparison between AYAs and adults yielded a 5-year relative survival of 90.6 (89.6–91.5) in AYAs and 89.1 (88.6–89.6) in adults, the difference being statistically significant (*p* < 0.007), but excluding the four atypical diagnostic groups from the standard population yielded a 5-year relative survival of 92.5 (91.5–93.5) in AYAs and 91.8 (91.3–92.3) in adults, the difference being statistically non-significant (*p* = 0.21).

**Table 1:** Relative survival by diagnostic group and age at diagnosis when diagnosed in 2000–2013 in Denmark, Finland, Norway or Sweden

ICCC-3 diagnostic group	Age at diagnosis	*N*	Deaths	Relative 5-year survival in % (95% CI)	*p*-value	Cancers/deaths
1.a Lymphoid leukaemias (ALL)	0–14	2088	191	90 (88–91)	<0.0001	
	15–24	406	94	73 (69–78)	–	2705/341
	25–34	211	56	70 (63–77)	0.39	
1.b Acute myeloid leukaemias (AML)	0–14	388	93	74 (70–79)	0.18	
	15–24	252	70	69 (63–76)	–	959/274
	25–34	319	111	62 (56–68)	0.09	
2.a Hodgkin lymphomas (HL)	15–24	1473	37	97 (96–98)	–	2709/86
	25–34	1236	49	96 (94–97)	0.06	
2.b non-Hodgkin lymphomas (NHL)	0–14	312	29	90 (87–94)	0.07	
	15–24	498	66	86 (83–89)	–	1770/203
	25–34	960	108	87 (85–90)	0.39	
3.b Astrocytomas	0–14	823	122	84 (81–86)	0.0017	
	15–24	512	105	76 (72–80)	–	2015/493
	25–34	680	266	55 (51–59)	<0.0001	
3.c Intracranial and intraspinal embryonal tumours	0–14	408	134	64 (59–69)	0.98	
	15–24	77	25	64 (53–77)	–	519/175
	25–34	34	16	38 (22–67)	0.08	
3.d Other gliomas	0–14	121	37	67 (59–76)	0.09	
	15–24	153	30	77 (70–85)	–	618/130
	25–34	344	63	78 (73–83)	0.86	
3.e Other specified intracranial and intraspinal neoplasms	0–14	325	14	95 (92–98)	0.21	
	15–24	427	11	97 (95–99)	–	1489/43
	25–34	737	18	98 (96–99)	0.68	
7.x Hepatic tumour—other^a^	0–14	30	12	45 (27–74)	0.39	
	15–24	26	13	30 (13–67)	–	136/69
	25–34	80	44	31 (19–50)	0.92	
8.a Osteosarcomas	0–14	172	40	73 (66–80)	0.11	
	15–24	176	57	64 (57–72)	–	397/111
	25–34	49	14	69 (56–84)	0.58	
8.c Ewing tumour and related sarcomas of bone	0–14	136	39	69 (61–77)	0.19	
	15–24	108	38	59 (50–71)	–	244/77
9.a Rhabdomyosarcomas	0–14	236	56	72 (66–78)	0.015	
	15–24	64	27	50 (38–66)	–	300/83
9.x NRSTS^b^	0–14	203	22	88 (83–93)	<0.0001	
	15–24	397	91	74 (69–79)	–	1207/231
	25–34	607	118	79 (75–82)	0.09	
a.c Malignant gonadal germ cell tumours	15–24	1896	37	98 (97–99)	–	6115/111
	25–34	4219	74	98 (98–99)	0.49	
b.d Malignant melanomas	15–24	1291	48	96 (94–97)	–	5564/251
	25–34	4273	203	95 (94–95)	0.20	
b.f.6 Carcinomas of breast	15–24	121	13	87 (81–94)		2708/382
	25–34	2587	369	82 (80–84)	0.12	
b.f.7 Carcinomas of cervix uteri^c^	15–24	182	19	89 (84–94)		1980/144
	25–34	1798	125	93 (92–94)	0.12	
All analysed cancers	0–14	5242	789			
	15–24	8059	781			31,435/3204
	25–34	18134	1634			

^a^“7.x Hepatic tumour—Other” is 7. “Hepatic tumours” excluding “7.a Hepatoblastoma” in the international Classification of Childhood Cancer, Third Edition (ICCC-3)^17^

^b^“9.x NRSTS” is “9. Soft tissue and other extraosseous sarcomas” excluding “9.a Rhabdomyosarcomas” in ICCC-3

^c^Numbers regarding “b.f.7 Carcinoma of cervix uteri” are based only on Denmark, Finland and Norway

**Fig. 1:**
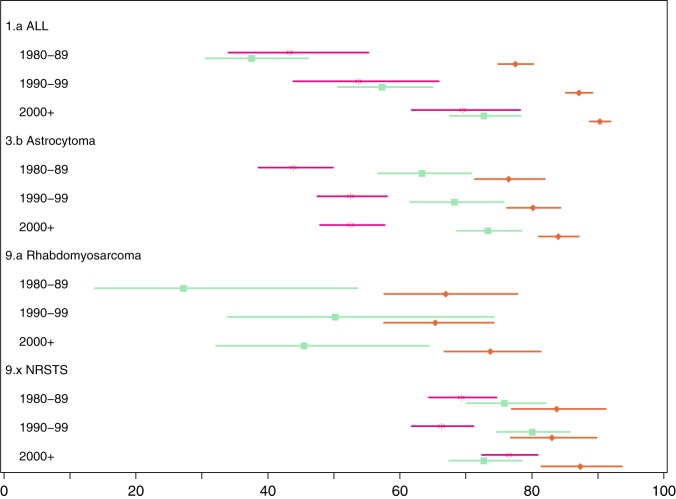
Relative 5-year survival in % with 95% confidence limits (*X*-axis) by diagnostic group, age at diagnosis and calendar period of diagnosis (*Y*-axis) when diagnosed in Denmark, Finland or Norway. Age at diagnosis 0–14 years (red), 15–24 years (green) and 25–34 years (magenta). Footnote: “9.x NRSTS” is “9. Soft tissue and other extraosseous sarcomas” excluding “9.a Rhabdomyosarcomas” in the international Classification of Childhood Cancer, Third Edition (ICCC-3)^17^

Figure 1 shows the historical development in 5-year relative survival for the four atypical diagnostic groups. They made up 1423/8059 = 18% of the investigated diagnostic groups in AYAs (Table 1). The results regarding the calendar period 2000+ presented here were very similar to the results obtained when Sweden was added to the material for all four diagnostic groups (Table 1). For the diagnostic groups of ALL and astrocytoma, all three age categories had substantial improvements in survival over time. For each period, survival was better in children than in AYAs, while survival was better in AYAs than in adults for astrocytoma patients and better or similar in AYAs compared with adults for ALL patients. The historical development in rhabdomyosarcoma survival was less clear cut. From 1980–1989 to 1990–1999, AYA survival made a leap while childhood survival stagnated, and the next decade saw increasing separation between the two with no overlap in confidence intervals. NRSTS survival was consistently better in children than adults, with no overlapping confidence intervals. AYA survival used to be in-between these two positions, but in 2000+ survival in adults improved dramatically and was better than in AYA.

## Discussion

Quality of life for cancer patients and AYA cancer patients in particular may be genuinely affected by a multitude of decisions regarding diagnosis, treatment, organisation of and access to healthcare. When measured against the ultimate hard measure of 5-year relative survival, we found that for the most common AYA diagnostic groups, AYA cancer patients in a contemporary Nordic setting were not in a disadvantaged position whether compared with children or with slightly older adults. Among recent European studies on AYA survival with multiple outcomes,^4,5,8,15^ the present study only overlaps with the EUROCARE-5 study based on data from 27 European countries.^5^ Patients in that study were diagnosed with cancer in 2000–2007, while the bulk of our study concerns patients diagnosed in 2000–2013. The relative 5-year survival reported here is noticeably better than what was reported in the EUROCARE-5 study for most diagnostic groups and age categories. This is probably due to both a better survival in the Nordic countries than in the rest of Europe and secular changes improving survival. This vindicates the present report as a harbinger of a more equal survival experience between age groups and better survival for all.

The historical development in survival following ALL and astrocytoma suggested neither survival nor rate of improvement in survival to be inferior in AYA compared with that in both the neighbouring age categories (Fig. 1). The historical development in survival following rhabdomyosarcoma suggests slight improvements both in children and AYAs. A comparison with adults was not available, but at least the improvement in survival in AYAs was similar to the improvement in survival seen in children (Fig. 1). NRSTS survival seemed stable in 1980–1999, then improving in children and adults in 2000+ without an equivalent improvement in AYA (Fig. 1). We think the most plausible explanation for this decrease in AYA NRSTS survival is a random change in case mix in this very heterogeneous cancer group. The incidence of NRSTS and rhabdomyosarcoma by period and age group was fairly stable and therefore not suggestive of misclassification between the two diagnostic groups (data not shown).

### Strengths and limitations

Ascertainment of the analysed cancer diagnoses and follow-up to death from any cause of the patients through the Nordic cancer registers and civil registration systems is virtually complete.^16^ The age range at diagnosis used to delineate AYA has varied considerably between studies.^5^ A lower age limit of 15 years has been common with a few exceptions, e.g. refs. ^8,15^ while the upper age limit has varied much more: from 19 to 49 years.^5,15^ The most recent large study of AYA mortality in Europe^5^ has used the age range 15–39 in accordance with proposals from the US National Cancer Institute^22^ as accepted by European Network for Cancer in Children and Adolescents.^5^ Our AYA age range was much narrower, 15–24 years, and for the adults we compared them with similarly younger, 25–34 years at diagnosis. Our definition of AYA makes especially the comparison with childhood cancer more relevant and makes the AYA group much more homogeneous with respect to behaviour, physiology and responsibilities. The flip side of that choice is of course that it diminishes statistical power. We have used the ICCC-3 childhood cancer classification^17^ to define the cancer categories for study, because our primary perspective has been the comparison of childhood and AYA patients, paediatric and adult treatment and an assumption or hypothesis that paediatric treatment schemes on average would benefit AYA patients more than contemporary AYA or adult treatment schemes, as previously exemplified by treatments for ALL and bone sarcoma.^9,13,14^ Both the ICCC-3 and the ICD-O-3 classifications are considered suboptimal for analysis of AYA cancer epidemiology; the ICCC-3 among other things lacking detail about the carcinomas that are very common in AYAs and the ICD-O-3 making distinctions between cancers based on topography that often seem irrelevant in AYA patients.^23^ Since the focus of this paper is on cancers that are common in both narrowly defined AYA patients and childhood patients, we have not found it constraining to stay within the confines of the ICCC-3. In another approach based on SEER data from 1973 to 2014, Liu et al. chose to sacrifice cancer-specific analyses to obtain detailed results by sex, age group and calendar year, reaching conclusions very similar to ours.^24^

Relative survival as traditionally calculated has been criticised for being unduly dependent on national background rates.^25^ However, as the mortality rates are very low in the analysed age span (<40 years) this should be only a theoretical concern. The data we analysed were aggregated according to several classification criteria from individual-level data into sums of follow-up time, events and expected events with given characteristics (age, sex, country, cancer, follow-up period and time interval since diagnosis). We did so for practical reasons to end up with data suitable for Poisson regression analysis,^19^ but primarily to provide an easy solution to data security issues allowing us to analyse all statistically sufficient data in one place jointly. The basic model was an additive hazard model combining a background expected death rate and an excess death rate due to cancer. Thus, this model could not accommodate relative survival larger than 100%. This did not turn out to be a practical problem either. The restriction that any given cell in these aggregated raw data should be based on at least five persons (Denmark) or three persons (Sweden) does not bias our results, under the assumption that the true excess hazard rates are the same in all four countries and both sexes, it merely decreases the precision of our estimates. For nine out of the 17 outcomes studied, not a single cell was missing from Denmark or Sweden. Since childhood and AYA cancers are generally rare diseases, it has been useful powerwise to conduct the analyses for the Nordic countries combined.

The main limitation of our study is a lack of information on background variables that may explain our findings based on cancer subtype, treatment, disease stage, organisation of healthcare provision etc. This can only be addressed properly in concerted international collaborative studies to obtain sufficient size and harmonisation for meaningful statistical comparisons. A major problem for such studies would be that AYAs participate so little in experiments and protocol trials.^10–12^

## Conclusion

The present study suggests meaningful differences in survival between AYA patients and either childhood or adult patients to be a thing of the past for most AYA cancer diagnostic groups in an affluent setting with free access to healthcare. This means that other measures of quality of treatment will have to take a more prominent role when assessing what works and what does not work in the quest for “personalised medicine” that fully acknowledges the different needs of different groups of patients according to their age. Stated differently, the observed 2.4% better overall survival in children than AYAs and 0.7% better survival in AYAs than adults in 13 of 17 cancer diagnostic groups investigated provides little evidence of low-hanging fruits or wrongfully neglected possibilities in AYA cancer treatment. The difference in survival may very well be real but is just as likely due to, e.g. a less fortunate stage distribution in AYAs than children, as systemic deficiencies in AYA treatment and diagnosis. It will take the aforementioned large concerted international collaborative studies to explore this further. Survival after ALL, astrocytoma, rhabdomyosarcoma and NRSTS was worse in AYAs than children. With the caveat that this may be explained perfectly well by case mix (e.g. by stage), it warrants initiatives to improve survival in AYAs after these cancers.^2,7,10,12–14,26,27^

## Data Availability

The datasets generated during and/or analysed during this study are not publicly available due to confidentiality reasons, but the sufficiently aggregated data used for analyses may be available from the corresponding author on reasonable request.
